# The efficacy of a nursing care and follow-up program for patients with heart failure: Study protocol for a randomized controlled trial

**DOI:** 10.1097/MD.0000000000023380

**Published:** 2020-12-04

**Authors:** Zhimin Zhang, Jincheng Bai, Yongmei Huang

**Affiliations:** a Department of Cardiovascular Medicine; b Department of Nursing, Changshan County People's Hospital, Zhejiang, China.

**Keywords:** heart failure, life quality, nursing care, protocol

## Abstract

**Background:**

Heart failure (HF) is one of the primary causes of the increasing public health costs, incidence rate and mortality of heart disease. As treatment options for the HF have evolved, people have a better understanding of overall burden of HF, resulting a more centralized method for the treatment of these patients with chronic diseases. At present, with the rapid progress of medical technology, the nursing mode must be updated accordingly. The objective of this trial is to investigate the effects of the program of nursing care and follow-up on life quality, self-care, and the rehospitalization of patients with HF.

**Method:**

This is a randomized controlled study to be carried out from November 2020 to March 2021 and was granted through the Ethics Committee of Changshan County People's Hospital (CCPH002376). The patients meet the following criteria will be included: the age of the patients is 18 years and above, and the functional classification is NYHA II or NYHA III. The patients with the following criteria will be excluded: patients who have received the by-pass surgery in the last 6 months; cancer patients are given radiotherapy or chemotherapy; patients with severe renal failure requiring dialysis; patients with chronic obstructive pulmonary disease who need ventilation; and patients with hearing or visual impairment. In our experiment, patient information scale, the life quality scale (The Left Ventricular Dysfunction Scale) and Self-Care of HF Index are utilized for the assessment. All the analyses are implemented with SPSS for Windows Version 20.0.

**Results:**

Impact of experimental programs on outcomes will be illustrated in the Table.

**Conclusion:**

We hypothesize that the nursing care conducted for the HF patients may improve the life quality and self-care.

**Trial registration number:**

researchregistry 6129.

## 1 Introduction

Heart failure (HF) is one of the primary causes of the increasing public health costs, incidence rate and mortality of heart disease.^[1,2]^ In the United States alone, more than 4 million people suffer from the congestive HF each year, and more than 550,000 cases are reported each year.^[3]^ About 1 to 3 percent of adults are diagnosed with the heart disease, and in their lifetime, 1/5 of them develop HF, and incidence rate increases with the age.^[4]^ In the natural process of HF, people will undergo the acute attacks and need hospitalization and emergency medical treatment. It is predicted that in the next decade, the number of people hospitalized for the HF will increase significantly, which will put more and more pressure on the system of health care.^[5,6]^ As treatment options for the HF have evolved, people have a better understanding of overall burden of HF, resulting a more centralized method for the treatment of these patients with chronic diseases.

In these methods, care is an important part, which is designed to help people who have experience with illness make changes in their behavior conducive to good health. At present, nursing practice is seeking to conduct the interventions based on evidence in the clinical settings, with particular emphasis on outcome assessment.^[7,8]^ The HF patients encounter great difficulties in their self-care. Lee et al^[9]^ have reported that if the patients with HF could regularly monitor their symptoms, the management of self-care could be refined and the rehospitalization rates of these patients could be decreased. At present, with the rapid progress of medical technology, the nursing mode must be updated accordingly. The objective of this trial is to investigate the effects of the program of nursing care and follow-up on life quality, self-care, and the rehospitalization of patients with HF.

## 2 Methods

This is a randomized controlled study to be carried out from November 2020 to March 2021. This trial is implemented in accordance with the SPIRIT Checklist for the randomized researches and was granted through the Ethics Committee of Changshan County People's Hospital (CCPH002376), and this trial was registered with research registry (researchregistry6129).

### 2.1 Randomization

This study includes 90 HF patients. A random number is assigned to all the patients through utilizing via using a random number table, and the allocation result is hidden in a random envelope. Patients are divided randomly into the study group and control group, each group is assigned 45 people. The patients with the following criteria will be excluded: patients who have received the by-pass surgery in the last 6 months; cancer patients are given radiotherapy or chemotherapy; patients with severe renal failure requiring dialysis; patients with chronic obstructive pulmonary disease who need ventilation; and patients with hearing or visual impairment.

### 2.2 Nursing Interventions

In control group, patients are given the standard nursing in the HF outpatient clinic. The doctor carries on the physical examination to the patient, then prescribes the medicine to patient, ultimately, arranges the appointments of outpatient follow-up. The questions of patients are answered by nurse-researcher.

In intervention group, the patients are given the education manual on the basis of the theory of self-care for HF. Moreover, they are also offered a set of magnetic induction instructions that they should pay attention to in their daily life. The patients are asked to attach magnets to their refrigerators so they could easily follow up on their daily visits and be alerted. After baseline data are taken from patients in intervention group, the patients are followed up by nurse researchers on the phone every 2 weeks for the period of 6 months. Nurse researchers carry out the physical examination for the patients, and provide the personal education and then tell the patients how to carry out some relevant daily monitoring. On the basis of the personal daily lifestyle, the prescribed medications can be adjusted by educational intervention. At the clinic, the patients need to bring the training materials with them. Their symptoms, for instance, the blood pressure, edema, taking additional diuretics as well as the levels of their exercise at home are examined. Through the provision of education manuals and personal education, we strive to improve the knowledge and awareness of the patients. By using the educational manuals designed via nurse researchers, magnet supports the telephone follow-up and educational materials to ensure monitoring of individual symptoms, adherence to treatment, and the identification of symptoms, in an attempt to develop the maintenance of self-care. Outpatient consultation and follow-up also contributes to the management of self-care.

### 2.3 Outcome measures

In our experiment, patient information scale, the life quality scale (The Left Ventricular Dysfunction Scale) and Self-Care of HF Index are utilized for the assessment.

### 2.4 Statistical analysis

All the analyses are implemented with SPSS for Windows Version 20.0. And the data are represented by using proper features, for example, percentage, standard deviation as well as mean. Independent samples *t*-test are utilized for comparison between groups. In order to compare the categorical variables between groups, we utilize the Chi-square test. For the significance level, the value of *P* needed to be less than .05.

## 3 Results

Impact of experimental programs on outcomes will be illustrated in the Table 1.

**Table 1:** Comparison of clinical outcomes between groups.

Variables	Study group (N = 45)	Control group (N = 45)	*P* value
Self-care maintenance			
Baseline to 2nd mo			
3rd to 4th mo			
5th to 6th mo			
Left Ventricular dysfunction scale			
2nd mo			
4th mo			
6th mo			
Total hospitalization cost			
Average daily hospitalization cost			
Rate of rehospitalization			
Complications			

## 4 Discussion

Due to low life quality, high medical costs and premature death, HF has become a kind of modern epidemic, which has brought huge economic and human burden to the community.^[10,11]^ The HF patients do not have enough self-care ability, and the rehospitalization rate for these patients is high.^[12,13]^ The maintenance of self-care includes the symptoms monitoring, adaptive therapy, as well as learning to recognize the relevant symptoms. It has been stated that identifying the symptoms is a significant step in managing the self-care of HF.^[14,15]^ In order to maintain an appropriate level of self-care, patients need professional nursing care, skills and knowledge. In the past decade, various interventions have been carried out in patients with HF. The effective nursing care and educational intervention for the patients can promote the development of positive health results.^[16,17]^ With the implementation of the follow-up plan and comprehensive care, the self-care abilities of patients with HF may be improved, and the hospitalization rate of these patients may be decreased.

## 5 Conclusion

We hypothesize that the nursing care conducted for the HF patients may improve the life quality and self-care.

## Author contributions

Jincheng Bai plans the study design. Yongmei Huang reviews the protocol and collects data. Zhimin Zhang writes the manuscript. All authors approve the submission.

**Data curation:** Jincheng Bai.

**Formal analysis:** Jincheng Bai.

**Funding acquisition:** Zhimin Zhang.

**Investigation:** Jincheng Bai.

**Methodology:** Yongmei Huang.

**Software:** Yongmei Huang.

**Writing – original draft:** Zhimin Zhang.
